# Supplementary material for: Improving understanding of disease control implementation research through a mooc with participants from low- and middle-income countries: Evaluating participant reactions and learning
Source: PLoS Negl Trop Dis. 2023 Mar 24;17(3):e0011139. doi: 10.1371/journal.pntd.0011139 (PMC10075403; doi:10.1371/journal.pntd.0011139)
Supplement: Attachment — Submitted filename: Reviewers comments.docx [file pntd.0011139.s001.docx]

**Reviewers comments**

**Details of how comments have been addressed**

Improving understanding of disease control implementation research through a MOOC with participants from low- and middle-income countries: Evaluating participant reactions and learning

|  | **Comment** | **Action taken** |
| --- | --- | --- |
|  | Key Review Criteria Required for Acceptance? |  |
| Reviewer 1 | Accept. Authors should include the CORE COMPETENCIES for IR | No action required |
| Reviewer 2 | The work should definitely made accessible to the wider audience of researchers, policy makers, and implementers in the PLoS NTD. The data is promising to show interesting results that can be used for the furthering of MOOCs and similar programmes.  The study design is appropriate, the population size sufficient in order to come up with the results. | No action required |
|  | However, the methods section has shortcomings as it  - lacks some of the information that is placed in the introduction  - does not describe what was being asked for in the exit survey – on which questions the results are being based on!  - the usage of datacol is not described  - the statistical analysis is not described | The Methods section has been reworked and added to. The following text has been added:  “The exit survey collected quantitative and qualitative data and consisted of 29 open-ended, multiple choice, and 5-point Likert-type questions (ranging from ‘to a very small extent’ to ‘to a very large extent’, ‘poor’ to ‘excellent’, ‘none’ to ‘very strong’). The survey captured participants’ demographics, expectations of, satisfaction with and evaluation of the MOOC, and understanding or IR before and after completion of the MOOC. The data reported on here is therefore self-reported by participants following completion of the MOOC.”  We can attach a list of the survey questions if the editor believes that will benefit the readers. |
|  | Other points:  - some results are shown in the methods section, whereas some of the methods are being delivered during the discussion of the results | Response number and rate moved to the results section.  The Methods section has been revised. |
|  | - when it comes to improvement, is there any baseline information from the participants (before – after, improvement from X to Y, etc?) | The results here are self-reported and there is therefore no baseline information. This is outlined in the *Limitations* section. Text describing the data as self-reported following completion of the MOOC has now been added to the *Methods* section (highlighted). |
|  | - aspects mentioned in the results are missing in the methods (see below) | The Methods section has been revised. |
|  | - methods need to be specific and clear, should in principle allow anyone to repeat the work, in this respect, for instance the sentence is both grammatically wrong, non understandable (lines 161-162). Also, the “need for ethics approval” cannot be “waived”. | Text added: “Respondents provided their consent to take part in the research through their participation in the survey.” |
|  | - was there any free text in the survey from which the results could gain insight? | Yes, but the focus of this article was to capture the numerical data. We have since published on open-ended questions and focus group data to glean further insights into participants experiences with the MOOC. |
|  | Throughout the text, it remains a bit vague whether there is reference to participants of the MOOC versus the survey. Altogether, it is difficult to address the review criteria given for the methods. | The authors have attempted to make it clear what results are being reported on at all times; The phrases “registrants”, “learners who completed the course/completers”, “responders” and headings such as “Results of the MOOC completion survey” are used to make the distinction clear. |
| Results | | |
| Reviewer 1 | Accept. Result may be presented in tabular form, if possible. | We have added Table 1 to address this comment |
| Reviewer 2 | The results match the indicated analysis plan, however it is difficult to follow the line of information throughout the paragraph:  - the order of listing should be consistent rather consistent than random – PhD – MD – MSc – Bachelor.  - the group of participants would need to be defined once clearly and should not be mixed up by the authors throughout the text  - a participation with more than 1% representation is an unclear criteria, in particular when the ones with 0.7% participation are also listed. | As above |
|  | The results section would benefit from (a) table(s) showing the demographics more clearly, with the countries of origin of the participants on the y axis, and the categories i) participated, ii) completed, iii) passed, [and possibly iv) completed the exit survey] on the x axis.  This would allow the deletion of abundant redundancies, i.e. lines 217-235 are repetitions.  Lines 237-238 are identical with 154-157. | We have added a table and deleted text using track changes. |
|  | As it comes to the reactions of the learners, there is no indication of the rating options they had, and hence any conclusion is difficult to assess, in particular when it comes to “improvement” of their IR knowledge. | See the amended Methods section which provides all of these details. |
|  | Methodology is missing for the following points that are outlined in the results and discussion:  - the “discussion forum” remains entirely unexplained hence is being shown as a result  - responders with and without certificate (271-275)  - occupations of the responders (276-280), how was this analysed?  - health officers (281 ff) – how was the grouping done (self-assessment?), which options were there and how was the distribution (see suggestion table)  - how was the “knowledge” or the “increase in knowledge” assessed? What does it mean “showed a strong/very strong knowledge in IR” – how was it measured, who assessed it, what is the difference between strong and very strong?  - what is referred to with “blending online course”? (385)  - what does this mean “helped ground the learning material”? and which “specific institutions” are meant (398 f.)  - not sure whether “globally” (403) conclusions can be drawn from  - the reader asks the question in line 408 why the data are not being “used to measure the specific improvement for knowledge in each competency before and after the MOOC training” – the title and abstract implied this was (intended to be) done? | The discussion forums were identified as contributing least to respondents’ learning - as stated in the Results section and linked to in Methods.  See Methods section that now addresses most of these queries.  Text added: “Descriptive statistics were calculated using Microsoft Excel.”  As stated in Methods,the responses were all self-selected. The ‘other’ option was also provided.  ‘Globally’ used here means that of the completers from the entire MOOC cohort - from 56 countries worldwide. |
|  | Some of the points should be shifted from the results to the discussion section (i.e. 286-291), whereas some of the discussion should be place to the results (i.e. 334-337).  Others should be shifted to the introduction, i.e. 375-376, 378-380. | Text from lines 281-291 has been moved to the Discussion section.  The results/numbers reported in lines 334-337 are repeated from the Results section and have been repeated in the Discussion section.  The authors are not sure what text from lines 375-376 belongs in the Introduction.  The following text has been added to the Introduction section: “Such findings are line with other studies regarding issues with discussion forums (e.g. Breslow et al., 2013; Hew & Cheung, 2014; Li & Canelas, 2019; Watson et al., 2016).” |
| Reviewer 3 | As said earlier, results are mainly quantitative and adequately presented. Using more maps and some tables/figures should improve the understanding of results. Yet, if the focus was on those who completed an exit survey, those data could gain more emphasis. Since social science researchers are critical members of IR teams, this profile and gender issues could be more explored (see line 233). It is unclear to me the text in lines 281-292. The authors had the intention to promote any modifications during the course time? Both health researchers and managers/officers have time limitations to accomplish the course tasks. Moreover, I am not sure if public health researchers consider MOOC certification crucial for career development. | These are good points raised, and many have been addressed in previous comments.  The authors want to provide a picture of the demographics of registrants, completers as well as the survey respondents as this all adds to our knowledge of PD MOOCs in LMIC countries and the types of people who access them, complete them and provide further information. |
| Conclusions | | |
| Reviewer 1 | Authors have made a novel effort in expanding knowledge through MOOC based training in a new and highly important area of Implementation Research for Public Health Programs. Very much needed for improving effectiveness of health programs in changing health outcomes and achieving sustainable development goal 3 - Universal Health Coverage. | No action required |
| Reviewer 2 | The conclusions should include  - that it is a natural explanation that the majority of participants “came from EN speaking countries” since the MOOC is in EN language! It should also be mentioned by the authors that the MOOC offer in other languages is provided with subtitles in those languages only but the videos are all in EN language, which may be a reason for many participants not completing the course. | This is discussed in the Discussion section where we state: “The majority of the registrants were from anglophone countries (87.2%).. The demographics of those who completed the course reflected those of the registrants.”  Not all videos were in English as some were in French.  This MOOC actually had a very high completion rate as stated in the following: “The completion rate of this MOOC was 30% which is higher than the 5-10% completion rate observed in high-income countries..”  We have not discussed the language in the Conclusions section as we instead focus on how the MOOC increased understanding of IR. |
|  | - the last section of the results section |  |
|  | It is unlikely that participants belong to a linguistic community (which is a homogenous group of people bound together by a common language) only when they come from same language speaking countries. | The overall grouping according to language was carried out because the findings (in the literature and the previous study) revealed that being able to comfortably converse in the same language was a barrier to participation in group discussions. As online forums tend to be a challenge with online learning generally, the step to delivering the MOOC in different languages was taken to increase inclusivity and accessibility. |
|  | The discussion refers to demographic profiles which are not explained before, 339-342 is a repetition. There is sudden mention of “employed responders” which brings in an entirely new dimension in the analysis not mentioned before. Also, for which is the certification “critical for their career development” – was that assessed? Why is this stated to be more critical for women than for men? Who are “active learners”? As opposed to “public health officers”?? | The text in lines 339-342 is paraphrasing information from the Results section.  “Employed responders” is used to contrast the high completion rate of Garrido (2016) with considerably lower completion rates generally found for MOOCs. Garrido’s study was of employed participants who were using MOOCs for PD purposes. In contrast, over ten percent of the IR MOOC participants were students.  The wording has been changed to “may be critical for their career development (10.7% versus 19%) should they wish to pursue IR-related research, further study or employment.” This is speculation by the authors as indicated.  The reason why certification may be more important for women is outlined in the previous sentence and referenced: “The fact that female students more often completed the course than males (58.8% versus 13.2%) may reflect the challenges that women generally face in their career development, such as being less likely to achieve the same level of career development and salary as men or face discrimination constraining opportunities in leadership roles, that may be amplified in LMICs (Halpaap et al., 2017; Liu et al., 2019; WHO, 2019a).” |
|  | The text of the conclusion, however, is mainly discussion. The 4 level method was never mentioned before and cannot be part of the conclusion. | This has been outlined in the Methods section and is now referred to in the Conclusion. |
|  | Same holds true for the introduction: “Many interventions remain under-utilised in many LMICs” – this is highly imprecise and subject to discussion in terms of correctness. In the beginning, IR would need to be explained for the reader. | The IR MOOC uses examples of this in relation to malaria and onchocerciasis.  IR is explained as: “Research on how to improve access, distribution and use of these tools (i.e. disease control interventions)”. |
|  | The Introduction should be set up to introduce the topic discussed in the paper to the reader, it somehow starts from somewhere and goes to somewhere else. Decision-makers and researchers are being mixed up within one sentence although they would need to be looked at very differently | Text added to clarify: “Targeting these distinct goups separately may lead to increased uptake in IR.” |
|  | The reference for the MOOC on IR with focus on IDPs is missing. | The reference has been added. |
|  | There are definitely too many redundancies to present this text to the audience of PLoS NTDs, starting from the introduction and carrying through to the end of the text. As a consequence, the Intro is way too long and parts of it belong to the Methods section. | This revision has minimised redundancies in the text. While the Introduction section is just over two pages in length now, the authors feel that the information provided is needed about IR, TDRs role in IR, training (or the lack of) in IR, and how MOOCs can assist.  The next section outlines the TDR MOOC in some detail so that readers can understand how the MOOC was structured and delivered and the experience that MOOC participants may have had. |
|  | The goal of the study, however, is not described except with one sentence under the header “Research Aims”. This needs to be restructured and expanded. | The Research Aims have been expanded. |
|  | The authors could state more clearly and specifically how these results help advance the understanding of the topic and will have public health relevance. | The following text has been added:  “The results from this research show that the IR MOOC can indeed increase understanding of the topic. An increase in IR knowledge may in turn strengthen IR capacity around noncommunicable diseases as evidenced through previous studies evaluating changes in professional behaviour (Launois et al, 2021) and organisations’ professional results (Ross et al. 2021) suggesting real-world benefits to public health outcomes.” |
|  | The limitations would need more detailed information, including some explanations of the lack of a control group, the bias of the responders and its influence on the results, also see aspects above. | In relation to the comment regarding bias, this is addressed in the following sentence: “Response bias may be present in those responding to the exit survey versus those who do not which may result in the positive results reported (Peytchev, Baxter, Carley-Baxter, 2009).” |
| Reviewer 3 | Much that was registered earlier fits for the Conclusion item. This piece is wellcome and can allow us to consider how to proceed to bring about IR to our empirical research on IDP. The text's title is "Improving understanding of disease control implementation research..." raises some doubts. The authors could make clear some points. Since we agree that IR is a multifaceted process, which specific topics were more important to explore? Saying differently, what was the main goal of the course? To introduce IR as a research strategy to reach better results on disease control? Or to go further in some topics helping researchers to design your own approach? Is it possible to answer what students can do now that we're not in the past? These are just some provocations to the authors. | The paper presents and evaluation of the IR MOOC using Kirkpatricks’ four-level model (Kirkpatrick & Kirkpatrick, 2006), where the first 2 points were addressed: 1) Reaction – the way learners react to the experience of the course; 2) Learning – the new knowledge, skills and attitudes they gained during the course. The second point is more directly linked to the title.  The authors are open to a title change, perhaps as suggested: “... Evaluating participant reactions and learning” |
| Editorial and Data Presentation Modifications | | |
| Reviewer 1 | Highly recommended for publication.  Include core competencies for Implementation Research  Data may also be presented in tabular form, if possible | The changes have been made as outlined above. |
| Reviewer 2 | Please see above for suggestions on  - a table  - shifting text elements between the headers introduction - methods - results - discussion - conclusion  - delete redundancies |  |
| Reviewer 3 | Maybe the authors could change the title of the article. | Changed to: “Improving understanding of disease control implementation research through a MOOC with participants from low- and middle-income countries: Evaluating participant reactions and learning” |
| Summary and General Comments | | |
| Reviewer 1 | MOOC based training on Implementation Research in health care is a novel approach to expand knowledge of implementation research. Building capacity of public health researchers and managers, policy and program managers in Implementation Research is the need of the hour across the developing countries.  Recommended for publication | No action required |
| Reviewer 2 | The article manuscript entitled “Improving understanding of disease control implementation research through a MOOC with participants from low- and middle-income countries” is a crucial contribution to the assessment of the effect of MOOCs in general, and in implementation research more specifically, and with LMICs in particular. The work should definitely made accessible to the wider audience of researchers, policy makers, and implementers in the PLoS NTD. The data is promising to show interesting results that can be used for the furthering of MOOCs and similar programmes.  However, the manuscripts needs a couple of clarifications and specifications to show what is intended to be shown. | Changes have been made to the manuscript according to reviewers’ comments and this has hopefully been addressed now. |
| Reviewer 3 | It is a good text that describes the first experience of MOOC training in implementation research. It would probably be appropriate to report that a substantial part of the students came from countries where the subject has already been discussed and experimented with in recent years. The big challenge is to take the course to regions where IR is still little known, but it is just as necessary, if not more.  The language will likely remain a challenge. Public policies and the structuring of health systems are also very different, and solutions must be local.  However, perhaps it is the beauty of IR: a continuous evolving process that needs to be revisited for each disease in each territory. Methodologies are essential as a guide to the path to be followed, but always need to be adapted to the lived world. | The main aim of this study is to examine how the IR MOOC cohort rated their understanding of IR knowledge. Further research should investigate the the points raises. |
| Reviewer #3: | The study presents its objectives and is well designed, the population is clearly described within an appropriate sample, although there may be no testable hypothesis. It should probably be explained a little more about the role and functions of MOOCs' training courses in the global learning strategies in the public health sector. We already have evidence of the profile of health professionals who enroll in this learning activity, especially in the global South. Actually, in some countries, MOOCs' learning experiences are relatively minor. Presencial encounters and practice experiments are fundamental since the health system's organizations are quite different. So, expectations and knowledge perceptions vary a lot. The statistics descriptions are fine but explain or advance little, in qualitative terms, about what was achieved given what the goal pursued. It seems evident that registrants came majoritarian from countries where some IR experiments already have  been taking place, which confers some bias to results. It should also be considered that the health themes involved in case studies can make difference in interest of enrolments: in which countries onchocerciasis and malaria are the most critical IDR? Again, IR is about methodologies but is crucially about diseases to the fought, the population involved, and the possible local strategies available. One question: who were the 110 participants that took part of the pilot study? All the comments above did not remove the merit of the MOOCs' experience described in the text. | These are excellent points raised and deserve further discussion.  As this is an exploratory study of whether or to what degree the IR MOOC contributed to participants’ self-perceived understanding of the topic and how they perceived the MOOC, the authors feel the analysis and level of detail presented in the paper is adequate.  In relation to the question of the 110 participants in the pilot, the manuscript refers to Launois et al. 2019 where we write: “Their feedback was then used to refine aspects of the contents and delivery, which included timing and workload, and issues with discussion forum engagement and language (Launois et al., 2019).” In this publication we state that: “Participants were a mix of programme managers, researchers and local health workers” and discuss other demographic information. |
