# Supplementary material for: Improving understanding of disease control implementation research through a mooc with participants from low- and middle-income countries: Evaluating participant reactions and learning
Source: PLoS Negl Trop Dis. 2023 Mar 24;17(3):e0011139. doi: 10.1371/journal.pntd.0011139 (PMC10075403; doi:10.1371/journal.pntd.0011139)
Supplement: Attachment — Submitted filename: Reviewers comments Dec 22.docx [file pntd.0011139.s002.docx]

**Reviewers comments**

**Details of how comments have been addressed**

Improving understanding of disease control implementation research through a MOOC with participants from low- and middle-income countries: Evaluating participant reactions and learning

|  | **Comment** | **Action taken** |
| --- | --- | --- |
| Methods | | |
| Reviewer #3 | Overall, the text became more evident after the review. As previously pointed out, it is an exploratory study; therefore, its innovative character and the importance of the topic discussion justify any minor limitations. | No action required |
| Results | | |
| Reviewer #3 | All of the items mentioned above have positively been achieved. | No action required |
| Conclusions | | |
|  | All of the items mentioned above have positively been achieved. | No action required |
| Editorial and Data Presentation Modifications | | |
| Reviewer #3 | It should be best explained the number of MOOC completers throughout the text: 1163 (Results)/546 (Table 1)/438(p.15). Indeed, what profile is the most important to authors: the completers or the survey participants? | 3858 registered for the MOOC  1163 earned a cert of completion  546 is the correct number of MOOC completers for which data was gathered for. (this has now been clarified in the manuscript)  438 of the survey respondents completed the MOOC (some respondents did not complete the MOOC)  574 responded to the survey |
|  | Attention to page 18 when it says that the new language versions will be available at the beginning of 2020/2021. | Text modified: “In response to this issue, TDR decided to translate the MOOC into all six United Nations languages. The Spanish and French versions were tested in 2018, and the Russian and Chinese versions were made available in 2020. The Arabic version was made available at the beginning of 2021.” |
| Summary and General Comments | | |
| Reviewer #2 | Review of revised version  The article manuscript entitled “Improving understanding of disease control implementation research through a MOOC with participants from low- and middle-income countries: evaluating participant reactions and learning” continues to give a crucial contribution to experiences with MOOCs for teaching and training purposes towards the research and implementation community, in particular in LMICs.  Some of the reviewer’s comments were addressed, others not or addressed half-heartedly. In principle the manuscript should be published but it is up to the editor to which extend reviewers investment on suggestions should be implemented before a final decision. | We have addressed the comments below in full and hope this alleviates Reviewer 2’s concerns. |
|  | The revised manuscript was re-sent to the reviewer. Unfortunately, the line numbering is not any more provided. | Unsure how to address this. |
|  | Some of the highlighted points were addressed:  - the title has been made more precise  - elements have been reassigned to the sections where the reader expects them (introduction, methods, results, discussions)  - the overall readability has improved  - the authors included a table as suggested which helps already | No action required |
|  | This table should, however, be improved – this table could stimulate the reader much more when (i) harmonized, i.e. always the same order (public health researchers, students,…) rather than randomly listed, (ii) information from the text could go into it, i.e. page 13, paragraph starting with “The highest education level”. If the information becomes too complex for one table, the content can be split into information for two different tables, some reflections on this could help improve and reduce the body to the text. | We have used the same order for all categories (highlighted). |
|  | Some of the other mentioned points were not addressed and lead to dissatisfaction:  - the methods are still scare and could be more detailed as the methods section should describe exactly so that the study could be repeated by anyone. However, some of the methodology is still scattered in other sections: i.e. page 7 paragraph starting with “The MOOC was hosted…” which is in introduction. If there was no free-text option in the survey, it should be mentioned, otherwise it would be an asset if some key citations could be added from these free texts. | The sentence: “The MOOC was hosted and run on the edX platform and offered by TDR.” is in the section describing the MOOC. As it was not part of the methods of this study, the authors have decided to keep it there.  The survey contained open-ended responses as stated: “The exit survey collected quantitative and qualitative data and consisted of 29 open-ended, multiple choice, and 5-point Likert-type questions”. We could include the survey questions as an appendix if the editor wishes.  The authors would gladly add citations, but we were limited by the word count. Other papers for this research include quotes from participants (from both surveys and interviews). We could quote these in the paper? |
|  | In particular, the introduction and discussion are still longish, show redundancies and are hence still a bit cumbersome to read, also page 14 paragraph starting with “The highest levels” is redundant to other passages. The beginning of the discussion is results and should go there. | We list levels of education for completers and respondents in the Demographics subsection of the Results section.  We have repeated certain figures in the Discussion section that were presented in the Results section as a way of summarising the data for the following discussion.  This has been modified to remove numbers:  “Registrants came from over one hundred countries from the six WHO geographical regions with most from the WHO African region. The majority of the registrants were from anglophone countries with significantly lower numbers from Spanish, French and Portuguese speaking countries, respectively.” |
|  | Minor points:  The title: it should most likely be plural to read: “evaluating participant reactions and learnings”? | We have used the noun ‘learning’ in this context as the abstract mass noun. |
|  | Some of the (unclear or misleading) formulations or imprecise wordings pointed out in the original review remained untouched, as are harmonization in order of listing (i.e. some of the “bachelor, master, PhD” listings). | The text has been read and edited for clarity. |
|  | page 13 bottom: change to read “other BRICS countries” | Added: “countries” |
